# The Static Magnetic Field Regulates the Structure, Biochemical Activity, and Gene Expression of Plants

**DOI:** 10.3390/molecules27185823

**Published:** 2022-09-08

**Authors:** Bogdan Saletnik, Aneta Saletnik, Ewelina Słysz, Grzegorz Zaguła, Marcin Bajcar, Anna Puchalska-Sarna, Czesław Puchalski

**Affiliations:** 1 Department of Bioenergetics, Food Analysis and Microbiology, Institute of Food Technology and Nutrition, College of Natural Science, Rzeszow University, Ćwiklińskiej 2D, 35-601 Rzeszow, Poland; 2 Laboratory of Physiotherapy in Developmental Disorders, Institute of Health Sciences, College of Medical Sciences, Rzeszow University, Al. mjr. W. Kopisto 2a, 35-959 Rzeszow, Poland

**Keywords:** magnetic fields, gene expression, enzyme activity, photosynthesis efficiency, plant components

## Abstract

The purpose of this paper is to review the scientific results and summarise the emerging topic of the effects of statistic magnetic field on the structure, biochemical activity, and gene expression of plants. The literature on the subject reports a wide range of possibilities regarding the use of the magnetic field to modify the properties of plant cells. MFs have a significant impact on the photosynthesis efficiency of the biomass and vigour accumulation indexes. Treating plants with SMFs accelerates the formation and accumulation of reactive oxygen species. At the same time, the influence of MFs causes the high activity of antioxidant enzymes, which reduces oxidative stress. SMFs have a strong influence on the shape of the cell and the structure of the cell membrane, thus increasing their permeability and influencing the various activities of the metabolic pathways. The use of magnetic treatments on plants causes a higher content of proteins, carbohydrates, soluble and reducing sugars, and in some cases, lipids and fatty acid composition and influences the uptake of macro- and microelements and different levels of gene expression. In this study, the effect of MFs was considered as a combination of MF intensity and time exposure, for different varieties and plant species. The following article shows the wide-ranging possibilities of applying magnetic fields to the dynamics of changes in the life processes and structures of plants. Thus far, the magnetic field is not widely used in agricultural practice. The current knowledge about the influence of MFs on plant cells is still insufficient. It is, therefore, necessary to carry out detailed research for a more in-depth understanding of the possibilities of modifying the properties of plant cells and achieving the desired effects by means of a magnetic field.

## 1. Introduction

The Earth’s magnetic field (geomagnetic field, GMF) is a natural component of the environment for all living organisms. The magnetic field is the primary environmental factor for plants on Earth. The study of the influence of static magnetic fields (SMFs) on biological systems has been of great interest for many years. The SMF is characterised by low unstable parameters relative to the other types of MFs, which facilitate its application in biological systems [1,2,3,4,5]. Researchers are focusing on finding a mechanism explaining such interaction and developing a technique by which it is possible to shape the biological activity of a cell. Due to their nature, MFs can easily penetrate tissues and directly affect cell function [6,7]. Different cellular components and organelles, including mitochondria, cell membranes, protein, and DNA, change their electromagnetic behaviour under SMFs, hence affecting various physiological and biochemical responses in the cells [6]. A weak field with ‘very small forces’ can change the speed of electron movement. The rotations of the molecules carrying the magnetic moment precession can significantly initiate the subsequent biophysical processes. They are involved in non-specific responses to MFs that are seen in systems with processes involving cell growth and gene expression in plants. The plasma membrane is the principal structural element of the cell directly exposed to the MF. Thus induced structural changes in the cell membrane may affect cell properties such as changes in the shape and size of cells, ion activation, and dipole polarisation in living cells [8,9,10,11,12]. The effects of the magnetic field (MF) on plants, fungi, and microbes can be elucidated by ion cyclotron resonance (ICR) and the radical-pair model. These two mechanisms also play essential roles in the magnetoreception of organisms [1]. Studies in the literature on the subject [13,14,15,16,17,18,19,20,21,22,23,24,25,26,27,28] report that MFs influence the physiological and biochemical processes of plants, enzymatic activity, cell production, protein biosynthesis, photochemical activity, and the content of bioactive components. In this way, this field affects metabolism and cell division, activates plant growth, and shapes the quality characteristics of plants. MFs may also play vital roles in the nutrient uptake capacity of plants [29,30,31,32,33,34,35,36,37,38]. They may affect the reduction in toxins in plants, thus increasing health safety. MFs influence the diffusion of biological particles in solutions through the Lorentz force mechanism or the Maxwell stress, as well as the biochemical processes involving free radicals [39,40,41,42]. Other studies also report a positive effect of MFs on the content of photosynthetic pigments, photosystem II performance (PSII), and an efficiency index based on light energy absorption [18,43,44,45,46,47,48,49,50,51,52]. MFs can also influence the content of reactive oxygen and nitrogen species as the molecules formed in many biological processes, and they can also increase antioxidant activity and thus reduce oxidative damage to plant cells [4,40,52,53,54,55,56,57,58,59]. The influence of MFs on the expression of plant genes has been documented by many researchers [27,42,57,60,61,62,63,64]. They found significant changes in the expression of genes that play essential roles in regulating metabolism, biosynthesis, and the cellular stress response. As part of the research, attempts have also been made to explain the mechanisms of MF interaction with biostructures. Thus far, such a mechanism has not been experimentally verified. Several solutions have been proposed in this regard, including the radical-pair mechanism, the tripartite mechanism, and the level-mixing mechanism based on quantum biophysics [65,66,67,68,69].

In contrast, Barbic [6] proposed several other possible mechanisms for activating ion channels based on the magnetocaloric effect, the mechanical deformation of the cell membrane by diamagnetic forces, and the Einstein–de Haas effect.

In recent years, scientists have increasingly researched the use of MFs to improve plant growth and overall productivity. The MF is a technique that can ecologically and cheaply induce new properties in plants and is also useful in terms of their use in food and pharmacology. It can also shape plant resistance to diseases and pests to increase productivity. Despite numerous studies, it is still an innovative area of research focused on laboratory tests [70]. This review provides a ranking of the existing knowledge and the latest reports on the impact and possibilities of using the MF. It presents numerous scientific achievements on the impact of the MF on photosynthesis, cryptochromes, biomass productivity, reactive oxygen species, nitric oxide content, enzyme activity, structure and cell growth, plant components, and gene expression.

## 2. Effect of MF on Photosynthesis, Cryptochromes, and Biomass Productivity

Photosynthesis is the basis of life on Earth, leading to the production of biomass by converting CO_2_ from the atmosphere and sunlight to release oxygen. It is equated with a high-energy process and is also associated with the high efficiency of energy transfer. Photosynthesis can provide multiple parameters related to the activity and productivity of plants [71]. Sunlight captured by the plant splits the water and extracts the electrons, boosting an electron to a high energy level in photosystem II (PSII). Subsequently, the electrons travel through the chloroplast’s electron transport chain to photosystem I (PSI). At the end of the chain, the electron is passed to NADP^+^ to create NADPH. A share of the released energy is used to pump hydrogen ions driving ATP chemical energy production [65,72,73]. The research conducted, among others, by Deamici et al. 2019 [46], Thomas et al. 2019 [36], and Sarraf et al. 2021 [74] showed a positive effect of MFs on the photosynthesis apparatus. Photosynthesis parameters in soybean seedlings, such as the maximum quantum yield Fv/Fm, the quantum yield of electron transport *ϕ* Eo = ETo/ABS, the relative amplitude of the I–P phase ∆ V (I–P), PIABS, the rate of photosynthesis Pn, and the performance index PI, increased under the influence of the magnetic field, which contributed to a higher level of light absorption efficiency [48]. The performance index (PI) provides information on the structure and function of PSII and the performance of specific membrane electron transport reactions [75]. Shine et al. [43] showed that the treatment of MF soybeans (150 and 200 mT) resulted in a significant increase in quantum yields and the performance index compared with control plants. The pre-treatment of the SMF also increased the concentration of active PSII reaction centres. These results are consistent with previous studies after the magnetopriming of soybean plants [43,52]. A study by Baby et al. (2011) [76] also showed an increase in the performance index influenced by the density of reaction centres in the chlorophyll, the exciton trapped on the absorbed photon, and the efficiency with which the trapped exciton can transfer the electron to the transport chain. As a result, the plants showed high efficiency in the photosynthesis process.

Cryptochromes (CRY1 and CRY2) are photoreceptors through which photosynthetic organisms receive blue light (B, 400–499 nm). They are involved in many aspects of plant growth and development, such as stem elongation inhibition, leaf unfolding, chlorophyll production and initiation of photosynthesis, and stress response [77,78,79,80]. Phosphorylation is a metabolic pathway in which, under the influence of blue light, energy is released upon the oxidation of reduced nucleotides and converted into ATP energy [77,81,82]. Activation of cryptochromes is observed in periods of illumination alternating with darkness. Hore and Mouritsen (2016) [83] explained the biological photoreceptor as a magnetoreceptor function based on the radical-pair hypothesis. The produced unpaired radicals interact with magnetic fields, resulting in a change in the interconversion of the flavin redox state, and thus, the biological activity of the plant may change. The studies by Ahmad et al. (2007) [84], Xu et al. (2014, 2015) [85,86], and Pooam et al. (2019) [87] showed that an MF (500 µT) enhanced the biological response of cryptochromes to the applied MF. Using pulsed lighting conditions, they observed the reactions of cryptochromes to the applied MF. They documented that the Arabidopsis seedling growth was inhibited by activating cryptochromes in response to blue light. The greater the biological activity of cryptochromes, the shorter the seedling hypocotyl was recorded in response to blue light. The MF could change the ratio of different redox states of cryptochromes and thus change their biological activity. Other researchers such as Yu et al. (2007) [88] and Burney et al. (2009) [89] confirmed that MFs influenced the activation of cryptochrome by blue light. A 500 µT MF enhanced the blue-light-dependent phosphorylation of CRY1 and CRY2, while a nearly zero MF reduced the phosphorylation. In contrast, MFs attenuated CRY1 and CRY2 dephosphorylation in the dark, while a nearly null MF enhanced dephosphorylation in the dark. The modification of the levels of Arabidopsis cryptochrome phosphorylation via magnetic fields to some extent influenced the functions of the cryptochromes.

**Figure 1:**
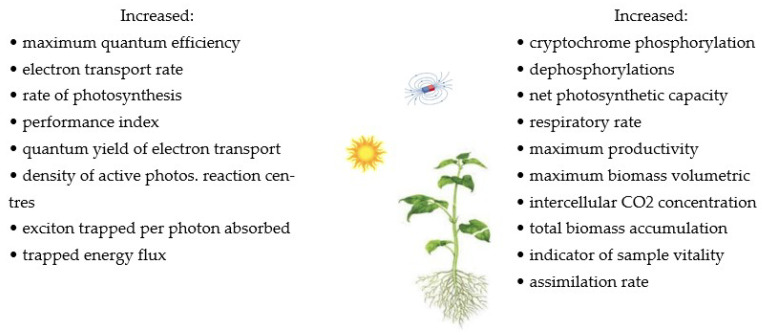
The summary of the effects of MFs on photosynthesis, cryptochromes, and biomass productivity [46,49]. Copyright *Bioresour. Technol*. **2019**, *292*, 121945. Copyright *Int. J. Mol. Sci*. **2021**, *22*, 9353.

The influence of MFs on cell growth and biomass concentration in plants was investigated [22,25,33,34,61,90]. The results of these studies showed that MFs increase biomass accumulation and vigour indicators, parameters related to germination, such as germination rate, water uptake, and seedling length. MFs cause cellular stress that can affect the growth and production of biomass. On the other hand, the continuous exposure of cells to MFs may promote the adaptation of cells to MF-induced stress, resulting in lower biomass production. The influence of MFs on biological systems can be treated as stimulating, in which two parameters play important roles in biomass productivity, exposure time (24 h/d and 1 h/d) and MF intensity (20, 60 mT, 250 mT). The methods using MFs and their effects on several plants in terms of photosynthesis, cryptochromes, and biomass productivity are summarised in Table 1 and Figure 1.

## 3. Effect of MFs at a Molecular Level

MFs may cause changes in the parameters of biochemical processes. They can regulate overall plant growth by influencing enzyme activity, metabolite transport, growth regulators, ions, and water. A lower concentration of MFs may stimulate the transport of carbohydrates and plant growth hormones to the distant growth zones of individual plant organs. The literature reports that MFs showed a positive effect on photosynthesis and the content of chlorophyll [2]. Geomagnetic fields can affect various enzymes. The activities of Ca^2+^/calmodulin-dependent cyclic nucleotide phosphodiesterase (20 μT) and cytochrome C oxidase (50 Hz) were altered by MFs [1,3]. MFs can also influence biological processes involving photochemical reactions. Scientists have identified the mechanisms of some changes in enzyme activity during exposure to MFs. MF effects are exerted by the inter-conversion of singlet and triplet rotatory states of the radical pair of biomolecules. Some enzyme reactions are sensitive, and their kinetics are affected by MFs [1]. MFs increase the content of auxins and the activity of enzymes that regulate the elongation of the plant cell wall [7]. They lead to an increase in catalase and peroxidase enzymes, the stimulation of reactive oxygen species, and changes in the activity of amylase and nitrate reductase in seeds [14]. An extremely low MF (0.2–0.3 μT) stimulated the activity of Na and K-ATPases, whereas a weak but moderate MF influenced the redox activity of cytochrome C oxidase. The treatment of 30 mT increased the esterase activity, whereas a 1 mT MF influenced the activity of horseradish peroxidase, and a strong MF (6 T) reduced the L-glutamate dehydrogenase and catalase activity, but 2 T substantially enhanced the activity of carboxydismutase. The strong MF also enhanced the activity of trypsin and ornithine decarboxylase [1]. MF affects the membranes and Ca^2+^ signalling in plant cells, and many magnetic effects in living organisms are probably due to the alterations in membrane-associated Ca^2+^ flux. Na channels are less affected than Ca^2+^ channels, and due to the changes in Ca^2+^ channels, the Ca content might be reduced in MF-treated plants. MF treatment in seeds induces changes in the protein and lipid profiles of harvested seeds [15].

## 4. Effect of MFs on Reactive Oxygen Species, Nitric Oxide Content, and Enzyme Activity

Many biochemical processes, such as photosynthesis and metabolism, are accompanied by the formation of radicals or radical anions, which are dependent on the magnetic field because they have unpaired electrons [39]. These radicals are also formed under the conditions of abiotic and biotic stress. They can damage cellular components such as lipids, proteins, and nucleic acids [97]. The accumulation of radicals can also lead to disturbances in gene expression, changes in the activity of certain enzymes, damage to membranes, and a reduction in the level of antioxidant hormones [57,98,99]. Studies have shown the effect of static magnetic field on the accumulation of reactive oxygen species (ROS) [17,40,41,42,45,51,57,58,63,64,98,100]. Oxidative stress has been investigated in several plant species such as cherry tomato, cucumber, lettuce, maize soybean, tobacco, and tomato. There was a significant increase in superoxide radicals and hydrogen peroxide after plant exposure to a magnetic field of 20–250 mT for an exposure time of 0.5–12 h depending on plant species. Studies have shown an increase in superoxide radicals from 35% to 100% and hydrogen peroxide from 8% to 104% depending on the variety and the used method. The concentration of the hydroxyl radical (.OH) in maize and soybean increased from 16% to 50% with an increase in the intensity of 100–200 mT MF. Generally, the magnetic field increases the mean concentration of radicals, especially in the metabolically active tissues of plant cells that contain free radicals. Other studies [48,51,52,58,76,100] in some cases showed a decrease in the value of ROS under the influence of MFs. This was the case for 30–45-day-old soybean and lettuce exposed to a high MF of 770 mT. The studies by Kataria et al. 2021 [48], Latef et al. 2020 [100], and Chen et al. [95] found that under the influence of MFs, the content of hydrogen peroxide and superoxide radicals decreased, while the content of nitric oxide radicals increased. In other studies [42,59], the simultaneous effect of MFs on the content of hydrogen peroxide and nitric oxide was demonstrated. The enzymes scavenging and protecting against reactive oxygen species (ROS) include superoxide dismutase (SOD), which is involved in the detoxification of ROS and the breakdown of superoxide radicals into oxygen and H_2_O_2_; catalase (CAT), which eliminates hydrogen peroxide H_2_O_2_ and disintegrates H_2_O_2_ into water and oxygen and the peroxidase family (peroxides isozymes); peroxidases (POX), which catalyses the degradation reaction of H_2_O_2_; guaiacol peroxidase (GPX), which acts as an active scavenger of reactive intermediate types of radicals and catalyses the reduction of H_2_O_2_ and HO_2_ to water and lipid alcohols; and ascorbate peroxidase (APX), which is the most extensively dispersed antioxidant enzymes using ascorbate as substrate; POD is another peroxidase that utilises guaiacol and pyrogallol as substrates for H_2_O_2_ detoxification [91,97,99,101]. In addition, antioxidant enzymes protect the plant which results in higher plant productivity. The main protective role against free radicals is to increase the activity of ROS-capturing enzymes. The first enzymes of the detoxification process are SOD and CAT, the activity of which significantly increased in plants treated with MFs, namely in cherry tomato, cucumber, lentils, maize, microalgae, radish, shallot, soybean, wheat, and lettuce [17,28,40,100,102,103,104,105,106,107]. The registered higher CAT activity was related to SOD, except for the research plant cells of algae and the germinating seeds of the soybean. The increased activity of antioxidant enzymes under the influence of stimulating magnetic treatments may indicate the alleviation of oxidative stress. Studies have shown a higher activity of SOD and CAT in the roots of lentils and maize plants than in their shoots, while the highest activity of these enzymes was recorded in the leaves of shallot and wheat plants [28,104,106,107]. The exposure of wheat seedlings to an SMF (30 mT) increased CAT activity and decreased APX and PO activities, which resulted in a 43% reduction in lipid peroxidation [56,108,109]. Other studies [54] showed an increase in SOD activity in MF-treated suspension tobacco cells (10 and 30 mT), with a simultaneous decrease in the activity of CAT and APX enzymes.

**Figure 2:**
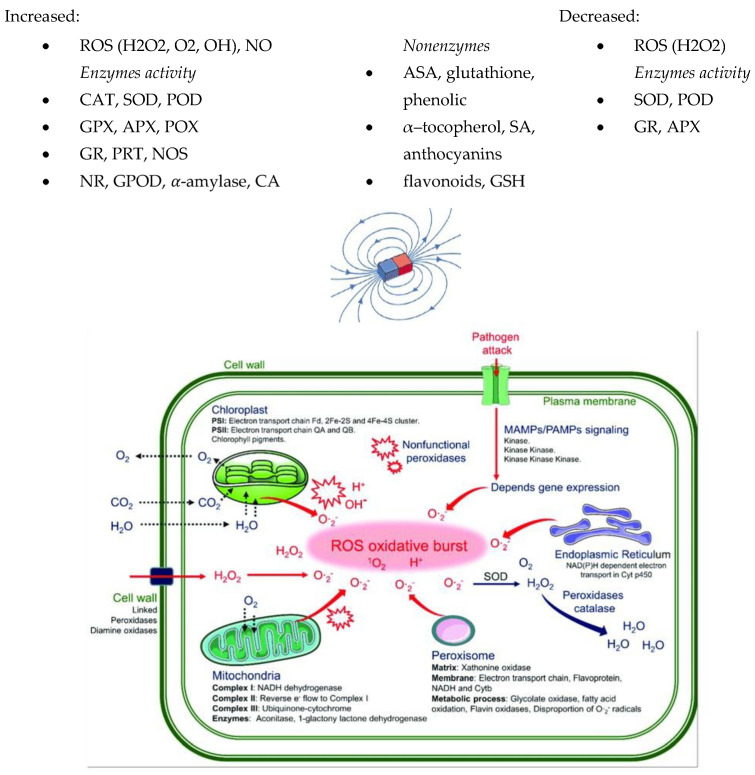
The summary of the effects of MFs on ROS, NO content, and enzyme activity [120]. Copyright Plants 2021.

The purification of ROS can also be achieved with non-enzymatic antioxidants. Non-enzymatic antioxidants such as flavonoid anthocyanins and carotenoids, which are abundant in several parts of plants, can contribute to H_2_O_2_ removal [97,110]. Flavonoids are a group of naturally occurring compounds showing antioxidant activity as secondary metabolites in plants. Ghanati et al. (2007) [101] and Jouni et al. (2012) showed a reduction in the total amount of phenolic compounds and flavonoids in basil and broad bean plants exposed to SMFs. Other studies showed an increase in ascorbate content in plants treated with an MF of 7 mT and a decrease in the value of this parameter at 150 mT [46,111]. MDA, a lipid peroxidation product, has been recognised as an indicator of oxidative damage. Electrolyte leakage is also generally considered an indirect measure of plant cell membrane damage [112]. Several studies showed the effect of an MF with an intensity of up to 100 mT on the increase in MDA content in plants, while for an MF of 600 mT, the index was significantly reduced [25,61,113,114]. The methods using MFs and their effects on several plants in terms of reactive oxygen species, nitric oxide content, and enzyme activity are summarised in Table 2 and Figure 2.

**Table 2:** Effects of MFs on reactive oxygen species, nitric oxide content, and enzyme activity.

Variety	Plant Species	Method	Effect	Reference
Basil (*Ocimum basilicum*)	12-week-old plants	30 mT SMF for 6 days at 5 h/day to plants	Decreased the activity of polyphenol oxidase (ap.24%) and phenylalanine ammonia-lyase (ap.68%) and phenolic compound content (ap.73%) in shoots; increased the amount of essential oils of methyl chavicol (46%)	[115]
Bean (*Phaseolus vulgaris* L.)	14-day-old plants	130 mT SMF within growing plants	Increased the guaiacol peroxidase activity by 44% in leaves but no significant changes in roots and shoots	[19]
Broad bean (*Vicia faba* L.)	Two-leaved plants	15 mT SMF for 8 days, each 8 h/day of plants	Increased the SOD activity (ap.30%) and the rate of lipid peroxidation (MDA ap.6%); decreased the total flavonoid content (ap.25%), and peroxidase and polyphenol oxidase activity (ap.18%)	[101]
Broad bean (*Vicia faba* L.)	8-day-old seedlings	30 mT SMF for 8 h/day of plants	Increased the content of hydrogen peroxide by 75% in the shoot and enzyme activity of CAT by about 100% in root and shoot	[110]
Cherry tomato (*Lycopersicon esculentum* L.)	Germinating seeds	50–150 mT SMF at 30 min and 1 h to seeds	Increased the radical content of superoxide (ap.100% at 4 h imbibition) and hydrogen peroxide (ap.60% at 24 h imb.) and antioxidant enzyme activities of SOD (ap.26%-36 h imb.), catalase (ap.36%-8 h imb.), POX (ap.78%-4 h imb.), APX (ap.150%-12 h imb) and GR (ap.50%-24 h imb.)	[40]
Cucumber (var. Barsati)	7-day-old seedlings	Pre-treatment of SMF 100 to 250 mT for 1, 2, or 3 h of seeds at imbibition time of	Increased the content of superoxide (40%), hydrogen peroxide (8%) and hydrolytic enzyme activity of b-amylase (51%), protease activities (13%), and the antioxidant enzyme activity of SOD (8%,), GR (77%), and CAT (83%)	[17]
Lentils (*Lens culinaris* L.)	15-day-old seedlings	Pre-treatment of SMF from 0.06 to 0.36 T for 5, 10, and 20 min to seeds	Increased the enzyme activity of APX by 210% and 350% in shoot and root, respectively (at 0.36 T, 20 min) but no significant changes in SOD	[104]
Lentils (10823 (ILL10823)	Shoots and roots of 7 days of plant growing	Pre-treatment of SMF 1–100 mT for 5–30 min to seeds	Increased the enzyme activity of SOD (to 170%), CAT (to280%), and APX (to 270%) in roots depending on the value of MFs; generally decreased the MDA enzyme (to 78%) in roots	[28]
Lettuce (*Lactuca sativa var. cabitat* L.)	14-week-old plants	Pre-treatment of 0.44, 0.77, 1 T for 1-3 h	Decreased the content of hydrogen peroxide ap.44%), superoxide (ap.44%), and malondialdehyde (31.7%) for 0.77 T at 1–2 h; increased the content of nitric oxide (ap.200%) and the antioxidant enzyme activities of SOD (ap.94%), POD (ap.900%), and GPX (ap.428%) for 0.77 T at 2 h; APX (ap.383%) and CAT (ap.750%) for 0.77 T at 3 h; the non-enzymatic of anthocyanins (257%), ASA (68.3%), GSH (69.7%), and α-tocopherol (165%) for 0.77 T at 3 h; and flavonoids (211%) and phenolics (355%) for 0.77, 1, and 0.44 T at 2–3 h, respectively	[100]
Lupin (*Lupinus angustifolius* L.)	14-day-old plants	0.2 mT at16 Hz and 50 Hz MF in growing plants	Increased the guaiacol peroxidase activity by 53% at 50 Hz in roots but no significant changes in shoots	[116]
Maize (*Zea mays* L.)	7–10-day-old plants	Pre-treatment of SMF 3 and 10 mT for 4 h of seeds	Increased the enzyme activities of SOD (178%, 432%), APX (90%, 100%), and CAT (160%, 468%) for plant (shoot, root) higher value at 3 mT	[104]
Maize (*Zea mays* L.) var. HQPM.1	8-day-old seedlings	Pre-treatment of SMF 200 mT for 60 min and 100 mT for 120 min to seeds	Increased the content of superoxide (31–57%), hydroxyl radical (26–39%), hydrogen peroxide (13–48%), and the enzyme activity of POD (10–58%), with a higher value at 200 mT; decreased the SOD enzyme activity (26–64%), with a lower value at 200 mT	[41]
Maize (*Zea mays)* var: HQPM.1	30-day-old plants	Pre-treatment of 100 mT for 2 h and 200 mT for 1 h to seeds	Decreased the antioxidant enzymes of SOD (43%) and POD (26%) and reactive oxygen species content of superoxide (26%) and hydroxyl (5%) in leaves at 200 mT	[14]
Maize (*Zea mays* L.) var. JM 216	45-day-old seedlings (leaves)	Pre-treatment of SMF 200 mT for 1 h to seeds	Increased the content of superoxide (52%), hydrogen peroxide (12%), α-amylase (76%), and protease activities (ap.3%) of seedlings; decreased the hydrogen peroxide (30%) in leaves	[45]
Maize (*Zea mays* L.) var. JM-216), soybean (*Glycine max* L.) var. JS-335)	8-day-old seedlings	Pre-treatment of SMF 200 mT for 1 h to seeds and inhibition time of 96 h	Increased the radical content of superoxide by 81% and 30% hydrogen peroxide −320% and 28%, and enzyme activity of α-amylase (ap. 70%) and 170% for maize and soybean, respectively, and protease s (ap. 6%), depending on inhibition time	[51]
Microalga (*Chlorella kessleri* LEB 113)	Plants with 10 days of cultivation	30 mT or 60 mT within 10 days of growing, exposure time 24 h or 1 h per day	Increased the antioxidant activity of methanol extracts by 77–217% at 60 mT for 1 h/d depending on the method	[25]
Microalgae (*Chlorella vulgaris*)	Algae cells in culture medium	10–50 mT SMF for 12 h to plant cells	Increased the antioxidant enzymes activities of SOD (124%), CAT (69%) at 50 mT, and POD (ap.50%) at 10–35 mT	[103]
Mung bean (*Vigna radiate*)	4-day-old seedlings	Pre-treatment of 600 mT SMF to seeds by conveyer belt	Increased the nitric oxide content (ap.32%, root; 36%, shoot), and the activity of nitric oxide synthase (ap.16, root; 25%, shoot); decreased the concentration of malondialdehyde (ap.56%, root; 8%, leaves), hydrogen peroxide (ap. 13%, leaves)	[95]
Parsley (*Petroselinum crispum* L.)	Plant cells after 6 and 12 h of treatment	30 mT SMF for 4 h	Increased the activity of CAT (38% at 6 h and 1500% at 12 h), and MDA indicator by ap.16% at 12 h; decreased the activity of APX by 30% and 70% after 6 and 12 h of treatment, respectively	[109]
Radish (*Raphanus sativus* L. var. radicula D.C.)	5-day-old seedlings	Treatment of 185–325 μT for 14 h to seedlings in light and darkness	Increased the activities of antioxidant enzymes of SOD (up to 135% μT), CAT (up to 135–150%) at 325–650 μT and soluble PO (up to 36–57%) at 185–310 μT, and malondialdehyde content (210%) at 325 μT; lowest value (110%) at 620 μT depending on the test	[117]
Shallot (*Allium ascalonicum* L.) bulbs	Plant of roots and leaves of 8, 12, and 17 days old (symplastic, apoplastic)	7 mT SMF for 17 days	Increased the antioxidant enzyme activities of GPOD (ap.33% for apoplastic), CAT (ap.40–50% for apoplastic and leaves), SOD (ap.20%, leaves), APX (ap.17%, leaves), the non-enzymatic activity of ascorbate (ap.39%), glutathione (ap.24%), the enzyme activities of glucose-6-PDH (30%), and glutathione reductase (ap. 25%) for leaves of 12–17 d old	[118]
Soybean (*Glycine max* L. Merrill)	Germinating seeds of 1–144 h	2.9–4.6 mT SMF at 2.2, 19.8, and 33 s to enzyme and seeds	Increased the antioxidant enzyme activities of SOD (130% at 19.8 s for 0–24 h) and CAT (20% at 19.8 s for 24 and 72 h) of root	[102]
Soybean (*Glycine max* L. Merrill J 357)	28-day-old plants	2.9–4.6 mT SMF at 2.2 and 19.8 s to seeds	Increased the peroxidase enzyme activity (36% at 19.8 s) and RNA concentrations (111%-2.2 s) for leaves	[119]
Soybean (*Glycine max* L. Merrill)	Approx. 15-day-old plants	20 and 30 mT SMF for 5 days, 5 h/d of plants	Increased the radical content of hydrogen peroxide (ap. 8–50%) and the enzyme activity of CAT (ap.16% for 2 d) at 30 m, contrary at 20 mT MF	[64]
Soybean (from Ayyub Agriculture Research Institute)	Seedling of early growth stage	Pre-treatment of SMF 50, 75, and 100 mT for 3 and 5 min to seeds	Increased the content of MDA (ap.40% at 50 mT for 3 min), ascorbic acid (ap.50–300% at 75 mT-3, 5 min), phenolics (ap.50%), and enzyme activity of PRT, α-AMY, SOD, CAT, and POD in the highest level of 75 mT at 3 and 5 min, and 50 mT and 100 mT at 3 min (over 50%)	[20]
Soybean (*Glycine max* L.) Merr. var: JS-335)	8-day-old seedlings	Pre-treatment of SMF 150 and 200 mT for 1 h to seeds	Increased the content of superoxide (33–75%), hydroxyl radical (16–50%), hydrogen peroxide (58–30%) in seedlings (embryo hypocotyl), and enzyme of POD (27%, cytosolic; 67%, wall-bound) at 200 mT; decreased ascorbic acid (53%, embryo; 37%, hypocotyl), SOD (12%, cytosolic; 27%, wall-bound), APOX (38%, hypocotyl) at 200 mT	[14]
Soybean (*Glycine max* L.) var. JS-335)	Seedlings growing within 5 days	Pre-treatment of SMF 200 mT for 1 h to seeds	Increased the content of hydrogen peroxide (77%), nitric oxide (42%), superoxide (35%), and enzyme activity of 𝛼-amylase (48%), nitrate reductase (178%), and protease (17%) in roots	[52]
Soybean (*Glycine max* L.) variety JS-335)	5-day-old seedlings	200 mT SMF for 1 h to seeds	Increased the radical content of superoxide (43%), hydrogen peroxide (104%), nitric oxide (50%), and enzyme activity of amylase (128%) NOS (75%), and NR (138%)	[42]
Soybean (*Glycine max* L.) Merr. var: JS-335)	30-day-old plants	Pre-treatment of 200 mT for 1 h and 150 mT for 1 h to seeds	Decreased the superoxide radical content by 16% in leaves at 200 mT	[43]
Soybean (*Glycine max* L. Merrill) var. JS-335	45-day-old plants	Pre-treatment of SMF 200 mT for 1 h to seeds	Decreased hydrogen peroxide content by 46%, and activity of antioxidant enzymes of SOD, APX, GR, and POD by 30–300% in leaves Increased α-tocopherol by 36%, ASA/DHA over 30% in leaves, and activity of nitrogenase enzymes in roots by 161%	[51]
Soybean (*Glycine max*) var. JS-335	45-day-old plants	Pre-treatment of 200 mT SMF for 1 h to seeds	Increased the activity of carbonic anhydrase (33%) in leaves and nitrogenase (151%) in root and nitric oxide (86%); decreased the content of superoxide (12%), malondialdehyde (14%), and proline (54%) in leaves	[59]
Soybean (*Glycine max*) var. JS-335	45-day-old plants	Pre-treatment of 200 mT SMF for 1 h to seeds	Decreased the hydrogen peroxide content (30%), activities of SOD (38%), POD (66%), and GR (60%) in leaves	[51]
Soybean (*Glycine max* L.) variety JS-335)	45-day-old plants	Pre-treatment of 200 mT SMF for 1 h to seeds	Increased the content of nitric oxide (ap.53%) and nitrate reductase activity (ap.33%); decreased the content of hydrogen peroxide (40%) and α-tocopherol (94%)	[48]
Tobacco (*Nicotiana tabacum* L. cv. Burley 21)	Plant cells	0.2 m T SMF up to 24 h	Increased the content of NO radical (25–100% for 8 h), hydrogen peroxide (25–108% for 18 h), and salicylic acid (9–30% within 8–24 h)	[63]
Tobacco (*Nicotiana tabacum* L. cv. Burley 21)	Plant cells	10 mT or 30 mT SMF for 5 days, from day 3 to 7 of subculture	Increased the activity of soluble peroxidase (61% at 10 mT), covalently bound peroxidase (46% at 30 mT), and decreased the ionically peroxidase activity fraction (ap.54% at 10 mT)	[53]
Tobacco (*Nicotiana tabacum* L. cv. Burley 21)	Plant cells	10 and 30 mT SMF for 5 days, 5 h each day	Increased the enzyme activities of SOD (87% at 30 mT) and decreased activities of CAT (70% at 30 mT) and APX (27% at 10 mT)	[53]
Tomato (var. Pusa Rohini)	Germinating seeds of 12 and 24 h	Pre-treatment of 100 mT SMF for 30 min	Increased the content of superoxide (38%), hydrogen peroxide (ap.100%), and antioxidant enzymes activities of catalase (3.7-fold) and ascorbate peroxidase (4.4-fold) at 24 and 12 h of imbibition, respectively	[57]
Wheat (*Triticum aestivum* L. cvs. Tekirdag and Selimiye)	28-day-old cultivars	Pre-treatment of SMF 2.9–4.7 mT at 2.2–19.8 s	Increased the enzyme activities of SOD (57%, 47%), POX (25%, 202%), APX (160%, 100%), CAT (190%, 100%) and FRAP value (40%, 43%) for Tekirdag cul. (leaf, root) higher value at 19.8 s	[107]
Wheat (*Triticum aestivum* L. cv. Kavir)	Approx. 4-day-old seedlings	30 mT SMF) for 4 days, each 5 h of germinated seeds	Increased the antioxidant enzyme activity of CAT (ap.70%) and decreased PO activity (ap.24%)	[108]
Wheat (*Triticum aestivum* L. cv. Kavir)	Approx. 3-month-old plants	30 mT SMF for 4 days, each 5 h of plants before harvest	Increased the activity of CAT (16-fold), radical scavenging capacity (13%) and decreased activity of PO (86%) and rate of lipid peroxidation of membranes (43%) of wheat seeds	[56]
Wheat (*Triticum aestivum*	100-day-old plants	Pre-treatment of max SMF of 50 mT by seeds or water passing	Increased the phytohormones content of gibberellic acid (76%), indole acetic acid (143%), and benzyl-adenine (212%), and decreased abscisic acid (22%) for seed +water	[69]

## 5. Effect of MFs on Structure and Cell Growth

**Figure 3:**
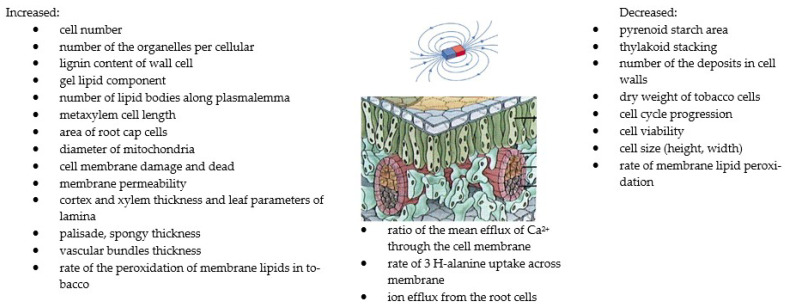
The summary of the effects of MFs on structure and cell growth [13,27,53,124,126]. Copyright https://brainly.co.id (accessed 23 August 2022).

The influence of MFs on the shape of the plasma and the plasma membrane of various biological systems has been studied [35,91,121,122,123]. Medium-intensity SMFs have a strong influence on the shape of the cell and the plasma membrane of various cell types. The extent of cell structure modification in response to SMFs significantly depends on the exposure time. Studies have shown a significant influence of MF plant treatment on biomass concentration [22,25,33]. The highest increases in this parameter, up to 83.2%, were obtained for an MF with an intensity of 60 mT and exposure time of 1 h/d. It was concluded that the application of a higher SMF intensity resulted in a greater amount of obtained biomass. The concentration of biomass results from the increase in the number of cells under the influence of MFs. Mroczek-Zdyrska et al. (2016) [19] showed a maximum cell growth of 79% at 130 mT, depending on the different phases of mitosis in the bean root meristem. Studies [96,124,125] showed a reduction in cell size (length and width) of up to 30% at 30 mT and the induction of longer metaxylem cells at 7 T with a time exposure of 30 h. Belyavskaya (2001) [126] studied the effect of a weak MF on changes in the structure and ultrastructural organisation of some organelles and meristematic cells of pea seedlings. Changes in the volume of the granular component of the nucleus decreased, the nucleolus vacuole appeared, and the shape changed to a more rounded one, compared with the control sample. The degree of alignment of cellular structures under the influence of SMFs depends on the intracellular composition [93]. Minor changes [93] were found in the ultrastructure of *C. kessleri* cells exposed to a 10 mT MF. Chloroplast and the area and number of starch granules significantly increased. Another study [96] showed internal changes in the stem and leaf structure parameters in tomato plants as affected by magnetised rehydration water and seed treatment with a 50 mT MF. These parameters of cortex xylem and lamina, and spongy and vascular bundles’ thickness increased, and higher values were recorded for magnetised water. Plant cell membranes are primarily exposed to stress, and changes in the membrane structure can cause intracellular modifications (Reszczyńska and Hanaka (2020) [127]). The loss of membrane integrity can alter the composition, structure, and function of plant cells. Selim et al. (2019) [35] found that cell membrane permeability was improved by a 50 mT MF. This parameter increased by about 29,97% when treating plants with magnetised grains, magnetised water, and the combination of magnetised grains and water, respectively, compared with the control. Reduced lipid peroxidation resulted in a decrease in electrolyte leakage and, therefore, reinforcement of membranes. These observations were confirmed by Payez et al. (2012) [108] for 4-day-old plants with a 30 mT MF. By contrast, Sahebjamei et al. (2007) [54] observed an inverse relationship, namely that exposure to MFs significantly increased the level of the peroxidation of membrane lipids of suspension-cultured tobacco cells, compared with the control. Ercan et al. (2022) [91] confirmed that MFs in the range of 20–250 mT caused cell membrane damage in the root tip cell of barley. Other studies [13,128] showed that MFs increased the rate of the efflux of calcium through the cell membrane and ions from the root cell. The magnetic field’s influence on living cells is on the cell cycle [63]. However, the intensity varies depending on cell type, the morphological modifications of cells, and treatment duration. It is assumed that the magnetic field affects the structures of cell membranes, thereby increasing their permeability, which, in turn, influences the various activities of the metabolic pathways. MF treatments can have a significant impact on reducing lipid peroxidation, which reduces electrolyte leakage and thus strengthens the membranes and improves plant growth. The methods using MFs and their effects on several plants in terms of the structure of plants, cell growth, and biomass productivity are summarised in Table 3 and Figure 3.

## 6. Effect of MFs on Plant Components

Biomass is rich in biologically active compounds such as peptides, polysaccharides, fatty acids, carotenoids, amino acids, etc. For this reason, it can be used in the production of biofuels, pharmaceuticals, and food supplementation [131,132,133].

Chlorophyll is the main photochemically active compound and plays a key role in the growth and adaptation of plants to various environmental conditions. SMF treatments significantly increased the chlorophyll content in barley, canola, chickpea, date palm, maize, maize, microalga, paulownia, soybean, sweet pepper, and wheat [23,31,36,45,48,91,94,134,135,136]. Static magnetic fields in the range of 100 mT and exposures for 240–360 min increased the content of photosynthetic pigments, chlorophyll, and carotenoids above 10% [134]. The combination of MF intensity and exposure time affects the pigment content. Long-term exposure to MFs may reduce pigment content [5,46]. The exposure time of 24 h, compared with 1 h, at the field intensity of 30 mT significantly reduced the content of chlorophyll and carotenoids in the leaves of microalgae. Similarly, an increase in MF activity from 30 to 60 mT resulted in a decrease in pigment content. Other studies [5] showed that the highest content values of chlorophyll and carotenoids in soybean were measured for 3 min 250 mT and were, respectively, almost 80% and 400% higher than those of the control group. On the other hand, an increase in the exposure time and MF intensity decreased the content of chlorophyll by 10% and 14%, and carotenoids by 32% and 57%, respectively. Research [23,32,35,137] has shown that the content of photosynthetic pigments is also significantly influenced by the magnetic water used for irrigation. Selim et al. (2019) [35] found that irrigated magnetic water increased the content of total chlorophyll and carotenoids by 40% and 50%, respectively, compared with the MF treatment of seeds. Moreover, MFs improve the absorption of the necessary elements needed for the formation of chloroplasts and chlorophyll, as characterised by paramagnetic properties [29,138]. The magnetic field stimulates cell growth and influences the biochemical composition of plants depending on factors such as the intensity and duration of exposure. Some studies showed a higher content of protein, carbohydrates, and in some cases, lipids compared with the control [22,25,37,91]. This fact proves that the MF application is a real alternative to the stimulation of lipid synthesis; however, it influences the synthesis of macromolecules in different ways. The use of 30 mT MF for 24 h increased the protein content in microalgae on average by 9%, while the highest carbohydrate content at the level of 45% was observed when using the higher field of 60 mT for 24 h compared with the control [46]. While Bauer et al. (2017) [25] recorded the highest increase in carbohydrate content by 14% for 30 mT, for the field intensity of 60 mT, the tested parameter significantly decreased. Treatment of plants with 30 and 60 mT MFs for 1 h also increased the lipid content by an average of 13% [139], although other studies [46] showed a decrease in the value of this parameter with an application time of 24 h. MFs have significant impacts on the content of other plant components such as proline, soluble sugars, amino acids, ferritin, and fatty acids [20,23,64,100,108]. The highest values of these parameters were recorded at field intensities of 30 and 100 mT, and 0.77 T.

**Figure 4:**
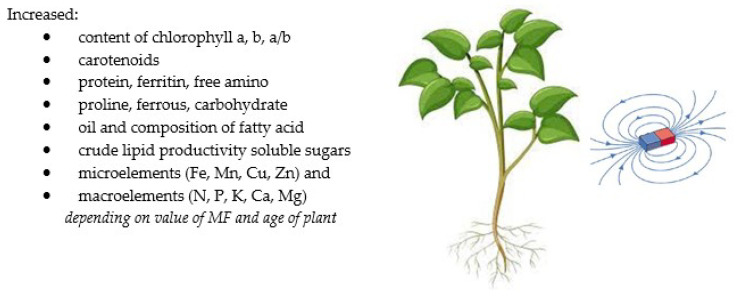
The summary of the effects of MFs on plant components [23,34,91,93,94,100,134,142].

Ferritin is attached to an ion channel and can influence the dynamics of ion transport and the changed movement of ions across the membrane [140]. Hozayn et al. (2016) [23] showed that the magnetic water irrigation of canola increased the content of unsaturated as well as saturated fatty acids and caused the decomposition of oil. In the group of unsaturated acids, linoleic and oleic acids dominated, while in saturated acids, palmitic and stearic acids dominated. Kataria et al. (2020) [58] showed a significant increase in the DNA and RNA content in plants exposed to a magnetic field, which could be caused by an increase in the expression of enzymes that play a role in shoot formation, chlorophyll biosynthesis, and peroxidase biosynthesis. The research of Asghar et al. (2016) [20] showed a higher soybean content of soluble and reducing sugars in the seedlings treated with a magnetic field, compared with the control. Studies have shown the effect of magnetic treatments on the uptake and accumulation of macroelements (N, P, K, Ca, and Mg) and microelements (Fe, Mn, and Cu) in the root and shoot of wheat plants (Selim and Selim) [35]. The maximum increase in uptake of the abovementioned minerals was recorded by the use of magnetised irrigation water compared with the MF seed treatment. Increases in the values of these elements ranged from 152% to 217%. The obtained results are consistent with those mentioned by Taimourya et al. (2017) [32] for strawberries and tomatoes. In contrast, studies on canola with the use of magnetic water showed a reduction in the content of elements such as N, Fe, and Zn and an increase in Mn and Cu compared with the control [23]. MFs may result in better water penetration through plant cell membranes, resulting in increased mineral solubility and better mineral absorption by plant roots [23,141]. Other studies have also shown an increase in the content of essential elements in plants treated with MF. There was an increase in the content of micro- and macroelements in the leaves of date palm plants under the influence of a 100 mT MF [134], while for microalgae, there was a decrease in the content of Fe and Cu microelements and an increase in Zn, Mn, Ni, and Ca for a 10 mT MF [93]. A study by Ercan et al. (2022) [91] showed a very significant reduction in the content of Mg, Ca, K, and P in the roots of the barley plant and rather their stabilisation in the leaves. On the other hand, the content of micronutrients significantly increased both in the roots and leaves of the plant. The methods using MFs and their effects on several plants in terms of plant components are summarised in Table 4 and Figure 4.

## 7. Effect of MFs on Gene Expression

**Figure 5:**
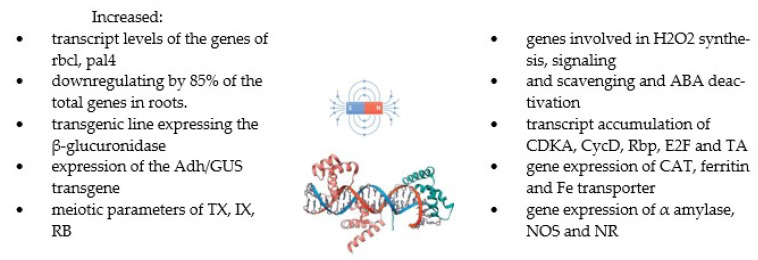
The summary of the effects of MFs on gene expression [27,42,57,62,63,64,128,143].

Magnetic fields influence DNA and RNA synthesis and cell proliferation and can cause changes in cellular metabolism and various cellular functions [59,143,146]. In doing so, they activate the cellular stress response as a protective mechanism that induces gene expression in the stress response. Paul et al. (2006) [129] found that MF T values induce the expression of the Adh/GUS transgene in Arabidopsis roots and leaves. Microarray analyses of 8000 genes showed that 114 genes were differentially expressed by more than 2.5-fold compared with the control sample. A static magnetic field of 30 mT increased the relative expression of the CAT and Fe transporter gene, which resulted in the enhancement of the total iron contents of the plants compared with the control. This induced the expression of the ferritin gene and an increase in ferritin content, which is involved in protection against oxidative stress [147]. Catalase is another main H_2_O_2_ scavenger, and the expression and activity of its gene increased in treated plants by MFs. However, a reduction in the gene expression of ferritin and CAT in a 20 mT SMF was observed. Ferrous content is superior to the expression of ferritin and CAT genes and is controlled by the expression of the Fe transporter gene. It can be processed by a magnetic field, which may have perturbed chemical reactions [129]. Some studies [42,148,149] showed a higher expression of the *α*-amylase gene and total amylase activity in seeds treated with SMFs, which resulted in increased seed germination and seedling vigour. This is mainly due to metabolic changes, including gene transcription, protein biosynthesis, and enzymatic activity [150]. The effects of MFs on gene expression have been investigated for very strong fields [129] and near-null and weak fields for GMFs [151,152]. A study by Dhiman and Galland (2018) [62] showed a significant effect of GMF reversal on the gene expression and growth of Arabidopsis seedlings. A similar effect on gene expression in null MFs was demonstrated by Xu et al. [153,154,155], and Agliassa et al. [151]. Another study [27] showed that in roots treated with a magnetic field, approximately half of the 359 downregulated genes changed more than two-fold compared with the control. The growth of chloroplasts was inhibited by a 600 mT SMF. Mohammadi et al. (2018) [63] and Okano et al. [111] showed that the exposure of tobacco cells to SMFs increased the content of reactive oxygen species and radicals (3–6 h of exposure), which modify proteins (through S-glutathionylation and S-nitrosylation) and thus regulate gene expression and activity proteins. Similar relationships were observed on germinated tomato seeds and magnetically treated soybean seedlings [42,156]. Anand et al. (2019) [57] investigated the expression of genes related to the synthesis, scavenging, and signalling of hydrogen peroxide under the influence of a 100 mT MF. The relative expression of genes involved in the production of hydrogen peroxide, i.e., amine oxidase (AO), superoxide dismutase (SOD1 and SOD9), and RACK 1 homologue (ArcA2) was significantly increased in treated seeds by MFs. Amine oxidase played a major role in the production of hydrogen peroxide, which regulates the expression of various genes involved in plant development. The methods using MFs and their effects on several plants in terms of gene expression are summarised in Table 5 and Figure 5.

## 8. Possible Mechanisms

Living organisms, including plants, generate various electric fields with which they are associated, such as membrane, electrical, functional, or flow potentials. Therefore, external MFs can influence plant development and metabolism through interactions [157]. In recent years, the following models explaining the mechanisms of the influence of MFs on biological systems have been proposed [18]: quantum oscillator and electronic cyclotron resonance quantum interference of bound ions and electrons, coherent quantum excitations, effects of torsion fields, free-radical mechanisms, parametric and stochastic resonance model phase transitions, etc. The radical-pair mechanism has been proposed to explain the effect of MFs on enzyme-catalysed reactions involving free-radical-pair intermediates [61,158,159]. Several studies [56,64,110] reported the influence of MFs on ROS production, the initiation of oxidative stress, the activity of enzymatic antioxidants, or the expression of their genes. It can also affect the singlet–triplet conversion of free radical pairs, which is driven by the internal magnetic fields produced by nuclear spins [74]. The energy involved in the recombination of radical pairs results from the interaction between the spins of unpaired electrons and the spin of adjacent nuclei; between the spins of the radical pair; and the interaction of the electron’s isolated spin and magnetic field (the Zeeman interaction) causing the direction of the electron’s magnetic moment to oscillate [61]. The magnetosensitive reactions of Arabidopsis plants were elucidated based on the radical-pair mechanism. Upon photoexcitation, a radical pair is formed, and the yield thereof depends on the direction of the MF. In this way, it allows the protein to act as a radical-pair-based magnetic sensor, which depends on the optimal radical-pair lifetime. The induced spin relaxation can also affect the magnetosensitive reactions of plants [159,160].

Binhi and Prato [65] developed the so-called molecular gyroscope mechanism. The essence of this mechanism is the rotation of large fragments of macromolecules or amino acid residues with distributed electric charges under the influence of MFs. The biological effect is related to the reaction yield, the number of gyroscopes that enter this reaction, or those that are in a state of equilibrium.

Vaezzadeh et al. (2006) [161] presented a theoretical model based on the oscillation of ferritin under the influence of MFs. The paramagnetic components of the cell include the concentrations of Fe, Co, and diamagnetic starch [162]. There is a theory that explains the increase in chloroplast content under the influence of MFs [142,163]. Chloroplasts contain Mn^2^^+^, which is a paramagnetic substance. Therefore, the MF energy may be absorbed, which affects the mobility and uptake of ions which play important roles in photosynthesis. Goldsworthy [164,165,166] presented the mechanism of changing the membrane potential and the permeability of the cell membrane under the influence of an electric field as a result of the selective removal of Ca^2^^+^ from the membrane and its replacement with other cations (mainly K^+^) [4]. Another model, the so-called ionic cyclotron resonance, was presented as a mechanism to explain the interaction between MFs and the ionic current in the plant cell membrane, which resulted in changes in ion concentration and osmotic pressure [15,135,167]. This mechanism is based on the interaction between the ions circulating in the plane perpendicular to the field and the MF. Binhi and Prato (2017) [65] presented a universal physical model as a mechanism for the interaction of MFs with the magnetic moments of unpaired electrons, paramagnetic ions (e.g., iron, copper, manganese), protons, and other particles. The disruption of the dynamics of the magnetic moment produces a biological effect at the physical and chemical levels.

## 9. Conclusions and Perspectives

The scientific achievements concerning the influence of MFs on the activity of plants, measured by the dynamics of changes in life processes, changes in the content of pigments and elements, and the structure of plants were reviewed. The various parameters of MF photosynthesis in plants were discussed in this review. Photosynthesis is a complex process, and the effects of magnetopriming have not been fully explored. The literature shows that MFs had a significant impact on photosynthesis efficiency. Research showed an increase in photosynthesis parameters in plants such as the maximum quantum efficiency, the electron transport quantum efficiency, the relative phase amplitude, the photosynthesis rate, and the efficiency index, which contributed to a higher level of light absorption efficiency. The research results presented in the literature review showed that MFs increase the biomass and vigour accumulation indexes, and thus have an impact on the plant yield.

In general, treating plants with SMFs accelerates the formation and accumulation of reactive oxygen species. This is related to the risk of oxidative stress. At the same time, the influence of MFs causes the high activity of antioxidant enzymes, which reduces oxidative stress. Research shows an increase in the activity of SOD, POD, and CAT enzymes of up to 300–400%, depending on the intensity of the MF, application time, and type of plant. The influence of MFs on the structure and development of cells was investigated in terms of the shape of the plasma and the plasma membrane of various biological systems. Medium-intensity (6 mT) SMFs have a strong influence on the shape of the cell and the structure of the cell membrane, thus increasing their permeability, which, in turn, influences the various activities of the metabolic pathways. Significant changes were recorded in the root meristem cells of plants exposed to MFs in terms of increases in their cell density and size. Changes in the ultrastructural organisation of some organelles, a decrease in the volume of the granular component of the nucleus, and the appearance of the nucleolus vacuole in comparison with the control roots were found. Static magnetic fields in the range of 10–100 mT and exposures for 30–360 min significantly increased photosynthetic pigments (chlorophyll, carotenoids). Studies also showed a higher content of proteins, carbohydrates, soluble and reducing sugars, and in some cases, lipids and fatty acid composition in plants under the influence of MFs, compared with the control. The use of magnetic treatments on plants influenced the uptake and accumulation of macroelements (N, P, K, Ca, and Mg) and microelements (Fe, Mn, and Cu) in the roots and shoots of plants. The influence of MFs on gene expression has been proven, and it depends on its intensity and application time. Researchers have shown that about half of the genes are regulated in roots treated with a magnetic field. A 30 mT static magnetic field increased the relative expression of the CAT and Fe transporter gene, which resulted in an increase in iron content in the plants compared with the control. Moreover, the greater expression of the ferritin gene, which is involved in the protection against oxidative stress and catalase as the main scavenger of H_2_O_2_, was shown. On the other hand, a higher expression of the *α*-amylase gene under the influence of MFs resulted in increased seed germination and vigour of seedlings. Research on the effects of magnetic fields on plants should be continued. This allows the development of a technique by which it will be possible to influence the course of biochemical processes related to plant metabolism and also influence enzymatic processes, chemical reactions, the structural system, properties of antioxidant plants, and the content of nutrition components. Owing to this research, it will be possible to discover the sense of sight and sensitivity of plants. There is a need to identify plant magnetoreceptors and study the cellular responses that convert pulses of a biophysical nature into quantum ones.

The current knowledge about the influence of MFs on living organisms is still insufficient, even more so because today’s research shows that many cellular properties can be modified through a static magnetic field.

## Figures and Tables

**Table 1:** Effects of MFs on photosynthesis and cryptochrome and biomass productivity.

Variety	Plant Species	Method	Effect	Reference
Barley (*Hordeum vulgare* L. cv. Tarm-92)	2-week-old seedlings	20, 42, 125, and 250 mT SMF to germinating seeds (four days) or seedling (two weeks)	Increased the maximum quantum efficiency of PSII (ap.6%) at 20 mT and electron transport rate (ap.38%) at 250 mT	[91]
Bean (*Phaseolus vulgaris* L.)	3-week-old plants	60 mT, 100 mT, and 160 mT for plant growing	Increased the relative change in photosynthetic apparatus efficiency (28.5%) and chlorophyll concentration (16.15) at 160 mT	[92]
Chickpea (Kabuli (Pusa 1053) and desi (Pusa 256)	70-day-old plants	Pre-treatment of 100 mT SMF for 1 h to seeds	Increased the rate of photosynthesis by 36%	[36]
Maize (*Zea mays* L.) var. JM 216	45-day-old seedlings	Pre-treatment of SMF 200 mT for 1 h to seeds	Increased the rate of photosynthesis (over 60%), performance index (65%), and the maximum quantum efficiency (ap.19%)	[45]
Maize (*Zea mays*) var: HQPM.1	30-day-old plants	Pre-treatment of 100 mT for 2 h and 200 mT for 1 h to seeds	Increased the maximum quantum yield (5%), the quantum yield of electron transport (14%), and the performance index (103%) at 200 mT	[43]
Maize (JM 216)	50-day-old plant	Pre-treatment of SMF of 200 mT for 1 h	Increased the maximum potential quantum yield (ap.6%), the quantum yield of electron transport (ap.20%), the performance index (42%), the net rate of photosynthesis (52%), and the nitrate reductase activity (ap.80%)	[45]
*Arabidopsis thaliana*	5-day-old seedlings	500 µT SMF to expose 10 min in dark and 5 min blue light, cycled for 90 min	Increased the cryptochrome phosphorylation (differential of 20%); cryptochrome responses to light were enhanced	[87]
*Arabidopsis thaliana* Columbia ecotype Col-4	Seedlings growing after 30, 60, and 90 min	500 µT SMF to expose 10 min in dark and 1 h blue light	Increased the cryptochrome phosphorylation of CRY1(7.617% after 60 and 90 min) and CRY2 (16.3% after 60 min) and dephosphorylations of CRY1 17.5% at 30 min) and CRY2 (18% at 15 min)	[85]
Microalga (*Chlorella kessleri*) (UTEX 398)	Plants growing in raceway pond and in flask cultures	10 mT SMF for plant growing	Increased the net photosynthetic capacity (210%) and respiratory rate (310%) and maximum photosynthetic efficiency (109%)	[93]
Microalga (*Chlorella fusca* LEB 111 C. fusca)	15-day-old plants	25 mT SMF for 24 h and 1 h/d	Increased the growth parameters of biomass by 32-85% (higher value for 24 h/d) depending on the environmental conditions	[94]
Microalga (*Arthrospira platensis* SAG 21.99)	10-day-old plants	30 mT SMF for 1 h/d and 24 h for plant growing	Increased the maximum productivity by 63% at 1 h/d and the maximum quantum yield (14%), the quantum yield for electron transport (23%), the trapped energy flux (11%), and the electron transport flux (15%) at 24 h/day	[46]
Microalga (*Chlorella kessleri* LEB 11)	Plants upon 10 days of cultivation	30 mT or 60 mT within 10 days of growing, exposure time 24 h or 1 h per day	Increased the maximum biomass volumetric productivity (59%) at 60 mT, 1 h/d	[25]
Mung bean (*Vigna radiate*)	4-day-old seedling plants	Pre-treatment of 600 mT SMF to seeds by conveyer belt	Increased the net photosynthetic rate (ap.16%), intercellular CO2 concentration (ap.18%), nitrogen (ap.6%) and chlorophyll content (ap. 10%)	[95]
Soybean (*Glycine max* L. Merrill) var. JS-335	45-day-old plants	Pre-treatment of SMF 200 mT for 1 h to seeds	Increased the rate of photosynthesis by 22.5% and total biomass accumulation (ap.50%)	[52]
Soybean (*Glycine max* L. Merrill)	45-day-old plants (roots, leaves)	Pre-treatment of SMF 200 mT for 1 h to seeds	Increased the rate of photosynthesis (by 22%), indicator of sample vitality (85%), the maximum quantum efficiency (32%), electron transport per leaf (50%), the activity of nitrate reductase (28%), transpiration rate (21%)	[44]
Soybean (*Glycine max*) variety JS-9560	45-day-old plants	Pre-treatment of 200 mT SMF for 1 h to seeds	Increased the maximum quantum yield (ap.10%), the performance index (ap.14%), and the rate of photosynthesis (ap.57%)	[47]
Soybean (*Glycine max* L.) variety JS-335	45-day-old plants	Pre-treatment of 200 mT SMF for 1 h to seeds	Increased the maximum quantum yield (ap.8%), the performance index potential (ap.140%), the quantum yield of electron transport (ap. 60%), and the rate of photosynthesis (ap.22%)	[48]
Soybean (*Glycine max*) var. JS-335	45-day-old plants	Pre-treatment of 200 mT SMF for 1 h to seeds	Increased the maximum quantum yield (ap.6%) and the density of active photosynthetic reaction centres (ap.9%)	[59]
Soybean (*Glycine max*) var. JS-335	45-day-old plants	Pre-treatment of 200 mT SMF for 1 h to seeds	Increased the maximum potential quantum yield (3%) and the rate of photosynthesis (32%)	[51]
Soybean (*Glycine max* L.) Merr. var:JS-335)	30-day-old plants	Pre-treatment of SMF 150 and 200 mT for 1 h to seeds	Increased the density of reaction centres in the chlorophyll bed (17%), the exciton trapped per photon absorbed (27%), and the efficiency of trapped exciton moving in the electron transport chain (16%) at 200 mT	[76]
Soybean (*Glycine max* L.) Merr. var: JS-335)	30-day-old plants	Pre-treatment of 200 mT for 1 h and 150 mT for 1 h to seeds	Increased the density of reaction centres (17%), the efficiency of light reaction (26%), the efficiency of biochemical reaction (16%), and the performance index (38%) at 200 mT	[76]
Soybean (*Glycine max* L.) Merr. var: JS-335)	30-day-old plants	Pre-treatment of 0-300 mT SMF for 30, 60, and 90 min to seeds	Increased the performance index (59%), the active reaction centre per cross-section (34%), the maximum quantum yield (6%), and the quantum yield of electron transport (14%) at 200 mT for 60 min	[43]
Tomato (*Lycopersicum esculentum* L. cv. Strain B)	70-day-old plants	Pre-treatment of max SMF of 50 mT by seeds or water passing through the magnetic funnel	Decreased the transpiration rate (45%) and increased the proline content (14%) for magnetised seed and osmotic pressure (14%) for magnetised water	[96]
Wheat (genotypes Kharchia 65 and HD 2967)	30-day-old plants	Pre-treatment of 50 mT SMF for 2 h to seeds	Increased the photosynthesis rate by 39% and 20% depending on the variety	[31]
Wheat (*Triticum aestivum* L. cv. Giza 168)	100-day-old plants	Pre-treatment of max SMF of 50 mT by seeds or water passing through the magnetic funnel	Increased the assimilation rate (24% for magnetised seeds and 57% for mag.water) and decreased the transpiration rate (46% mag.seed + water)	[35]

**Table 3:** Effects of MFs on structure and cell growth.

Variety	Plant Species	Method	Effect	Reference
Arabidopsis	7-day-old plants	With different directions of 300 mT or 600 mT within after 3 d of seed germination to 7 days of growing	Increased the meristematic cortex cell number (ap. 12%) and root meristem size (ap.10 %)	[27]
Barley (*Hordeum vulgare* L. cv. Tarm-92)	2-week-old seedlings	20, 42, 125, and 250 mT SMF to germinating seeds (four days) or seedlings (two weeks)	MFs induced cell membrane damage in roots Increased the cell membrane damage in the root tip cells at all MF strengths	[91]
Bean (*Phaseolus vulgaris* L.)	14-day-old plants	130 mT SMF within 14 days of growing plants	Increased the number of cells in the metaphase and telophase stages, by 23% and 79%, respectively	[19]
*Catharanthus roseus* (*Vinca rosea*, or *Madagascar periwinkle*)	Cell suspension cultures, intact cells, and their protoplasts	302 mT SMF for 0–220 min	Increased the force to regenerating protoplasts by 3.6-fold but no significant changes in the elasticity and diameter of intact cells	[114]
Lupin (*Lupinus angustifolius* L.).	14-day-old plants	0.2 mT at16 Hz and 50 Hz within growing plants	Increased the number of cells in the prophase stage (4–18%) and metaphase (20%), anaphase (23%) at 16 Hz and decreased by 18% (metaphase), 23% (anaphase), and 16% (telophase) at 50 Hz MF	[19]
Maize (*Zea mays* L., Pioneer HI-Bred)	2-day-old seedlings	7 T MF for 10–30 h	Increased the metaxylem cell length (ap. 20%), and area of root cap cells (54%) at 30 h Decreased the cell size (11%, height; 32%, width) and cell number (42%)	[124]
*Chlorella kessleri* (UTEX 398)	Plants growing in raceway pond and in flask cultures	10 mT SMF for plant growing	Increased the fatty acid composition of SFAs15:0 (20%) and MUFAs18:1n-7 (41%); increased the ultrastructure parameters of chloroplast area (31%), chloroplast starch granule area (148%), thylakoid area (41%), and starch granule number (176%); decreased pyrenoid starch area (54%), max. thylakoid stacking (33%)	[93]
Microalgae (*Chlorella vulgaris* L.)	Algae cells in culture medium	10–50 mT SMF for 12 h to plant cells	Increased the lipid peroxidation expressed (TBARS content) by 36% at 35–50 mT	[103]
*Chlorella kessleri* LEB 113	Plant upon 10 days of cultivation	30 mT or 60 mT within 10 days of growing, exposure time 24 h or 1 h per day	Increased the biomass concentration by 83.2% at 60 mT, 1 h/d	[25]
*Chlorella fusca* LEB 111	15-day-old plant	30 mT or 60 mT at 24 h or 1 h/day	Increased the biomass concentration by 27% for 30 mT at 1 h/d for 8–11 days and 45% for 60 mT at 1 h/d for 9–12 days	[22]
Pea (*Pisum sativum* L.)	3-day-old seedlings	0.5–2 nT MF for 3 days	Increased the number of organelles per cellular section (12%), the diameter of mitochondria (1.5–2-fold), and the number of lipid bodies along plasmalemma; decreased the number of deposits in cell walls	[126]
Spinach (*Spinacia oleracea* L.)	Plant plasma membrane vesicles	27 to 37 μT SMF for 30 min	Increased the ratio of the mean efflux of Ca^2+^ through the cell membrane (ap. 15%) at 30 to 32 μT	[129]
Spirulina sp. LEB 18	15-day-old plant	25 mT SMF for 24 h or 1 h/day of seedlings	Increased the biomass concentration by 16% during period (8–12 d) application of MF for 24 h in greenhouse	[33]
Tobacco (*Nicotiana tabacum* L. cv. Burley 21)	Plant cells	0.2 m T SMF up to 24 h	Decreased the dry weight of tobacco cells up to 75% for 3–12 and 24 h; decreased the cell cycle progression, a new cell cycle delayed 6 h	[63]
Tobacco (*Nicotiana tabacum* L. cv. Burley 21)	Plant cells	10 mT or 30 mT SMF for 5 days and 3 to 7 days of subculture	Increased the lignin content of wall cells (ap. 17%) and dead cells (ap. 100%) and decreased the size of cells (24–30%) and cell viability (ap. 21%) higher value at 30 mT	[53]
Tobacco (*Nicotiana tabacum* L. cv. Burley 21)	Plant cells	10 and 30 mT SMF for 5 days, 5 h each day	Increased the rate of the peroxidation of membrane lipids of suspension-cultured tobacco cells (ap. 33%)	[54]
Tomato (*Lycopersicum esculentum* L. cv. Strain B)	70-day-old plants	Pre-treatment of max SMF of 50 mT by seeds or water passing through the magnetic funnel	Increased the stem structure parameters of the cortex (5%) and xylem (5%) thickness for magnetised seed and leaf parameters of the lamina (11%), palisade (10%), spongy (6%), and vascular bundles (19%) thickness for magnetised seed and water	[96]
Tomato seeds (*Solanum lycopersicum* var Heinz H1439)	Plasma membrane	126 and 208 mT SMF	Increased the gel lipid component by 481%, protein component by 76%, and decreased fluid lipid component by 60% at 208 mT	[130]
Vicia faba	11-day-old seedlings	10, 100 uT or 1 mT AMF for 40 min to seedling root	Increased the rate of 3 H-alanine uptake across the membrane (40–92% at 100–10 uT 50 Hz) and ion efflux from the root cells (ap. 22% at 100 uT, 60 Hz)	[13]
Wheat (*Triticum aestivum* L. cv. Kavir)	Approx. 4-day-old seedlings	30 mT SMF for 4 days, each 5 h of germinated seeds	Decreased the rate of membrane lipid peroxidation (26%) and membrane electrolyte leakage (ap. 6%)	[108]
Wheat (*Triticum aestivum* L. cv. Giza 168)	100-day-old plants	Pre-treatment of max SMF of 50 mT by seeds or water passing through the magnetic funnel	Increased the membrane integrity (membrane permeability) percentage by 29% for magnetised seeds and 97% for magnetised water	[35]

**Table 4:** Effects of MFs on plant components.

Variety	Plant Species	Method	Effect	Reference
Barley (*Hordeum vulgare* L. cv Tarm-92)	2-week-old seedlings	20, 42, 125, and 250 mT SMFs to germinating seeds (four days) or seedlings (two weeks)	Increased the content of chlorophyll a and b, by about 35% and 18%; soluble protein in roots by 122% at 250 mT; chl a/b by 23% at 42 mT; and the microelement content (Fe, B, Cu, Mn, Zn, and Mo) of the leaves and roots up to 900%; decreased the content of carotenoids by 33% at 42 mT and macroelements (Mg, K, P, and Ca) by up to 800% in roots	[91]
Bean (*Phaseolus vulgaris* L.)	14-day-old plants	130 mT SMF within growing plants	No significant changes in pigment chlorophyll a, b, a + b, and carotenoid content	[19]
Canola (var. Serw-6)	180-day-old plants	Irrigated magnetic water by SMF of max 60 mT used during the growing season	Increased the content of chlorophyll a and b, carotenoids by about 13%, oil by 14.3%, and composition of fatty acid (stearic acid by 16%, oleic acid by 140%); decreased the element contents of N (17.3), Fe (6.7%) and Zn (17.7%) and increased Mn (9.1%) and Cu (28.6%)	[23]
Carrot	7 and 14-week-old seedlings	Pre-treatment of SMF 500 mT and 1 T for 3, 6, and 12 min to seeds	Decreased the mineral content of Cu, Fe, Mg, Mn, and Zn in the range of 37–52% at 1 T for 3 min and increased Na content by 44% at 500 mT for 12 min	[38]
Chickpea (Kabuli (Pusa 1053 and desi (Pusa 256)	70-day-old plants	Pre-treatment of 100 mT SMF for 1 h to seeds	Increased the total chlorophyll by 43–50% depending on the variety	[36]
Date palm (*Phoenix dactylifera* L.)	15-day-old seedlings old	10, 50, and 100 mT SMF at 30, 60, 180, 240, and 360 min to seedlings	Increased the content of chlorophyll a (ap. 180%) and b (ap. 150%) and carotenoids (ap. 100%) for 100 mT at 240–360 min Increased the mineral content of Mn, Fe, Zn, Ca, Na over 100% and Mg, K over 30% for 100 mT at 360 min in leaves	[134,142],
Lupin (*Lupinus angustifolius* L.)	14-day-old plants	0.2 mT at16 Hz and 50 Hz within growing plants	Decreased the content of chlorophyll a (82%) and b (74%), and carotenoids (64%) at 50 Hz, with no significant changes in protein content	[116]
Lettuce (*Lactuca sativa* var. cabitat L.)	14-week-old plants	Pre-treatment of 0.44, 0.77, 1 T for 1–3 h	Increased the content of chlorophyll a (ap. 400%) for 1 T at 1 h, carotenoids (ap 200%) for all, proline (489%) and soluble proteins (208%) for 0.77 T at 2 h, soluble sugars (102%) and free amino (144) for 0.44 T at 3 h	[100]
Maize (*Zea mays* L.)	7–10-day-old plants	Pre-treatment of SMF 3 and 10 mT for 4 h of seeds	Decreased the content of protein by 16% for shoots and 41% for roots; higher value at 3 mT	[143]
Maize (*Zea mays* L.) var: HQPM.1	30-day-old plants	Pre-treatment of 100 mT for 2 h and 200 mT for 1 h to seeds	Increased the content of total chlorophyll (16%) and total carotenoids (16%) at 200 mT	[144]
Maize (*Zea mays* L.) var. JM 216	45-day-old seedlings	Pre-treatment of SMF 200 mT for 1 h to seeds	Increased the chlorophyll content of a (26%) and b (83%)	[45]
Microalga (*Chlorella kessleri* UTEX 398)	Plants growing in a raceway pond and in flask cultures	10 mT SMF for plant growing	Increased the content of carbohydrate (8.5%), protein (8.7%), chlorophyll a (15%) and b (64%), and metal components of Ca, Zn, Mn, and Ni by 88–242%; decreased content of antioxidants (35%), and metal content of Fe and Cu by 30%	[93]
Microalga (*Nannochloropsis oculata*)	Approx. 7-day-old plants	20 mT,-40 mT SMF within 7 days of plant growing	Increased the crude lipid productivity by 65% and specific growth rate by 22% at 20 mT	[34]
Microalga (*Chlorella fusca* LEB 111, C. fusca)	15-day-old plants	25 mT. SMF for 24 h and 1 h/d	Decreased the total chlorophyll content by 12–33% and increased by 130–2058% for uncontrolled and control conditions, respectively, depending on time exposure; increased the protein content by 32.7% at 1 h/d in control conditions	[94]
Microalga (*Spirulina* sp. LEB 18)	15-day-old plants	25 mT SMF for 24 h or 1 h/of seedlings	Increased the chlorophyll a by 137.7% at 15 d in the chamber at exposure time of 24 h/d	[33]
Microalga (*Arthrospira platensis* SAG 21.99)	10-day-old plants	30 mT SMF for 1 h/d and 24 h for plant growing	Increased the content of carbohydrates by 21% at 1 h/d; decreased the content of protein t (18%), phycocyanin (26%) and chlorophyll a (27%) at 24 h/day in control conditions	[46]
Microalga (*Tribonema* sp.)	25-day-old plants	30 mT of SMF in plant growing	Increased the content of protein (6–48%), carbohydrate (4–15%), and oil (20–54%) depending on temperature	[37]
Microalga (*Chlorella kessleri* LEB 113)	Plants of 10 days of cultivation	30 mT or 60 mT within 10 days of growing, exposure time 24 h or 1 h per day	Increased the content of protein (8.9% at 30 mT-1 h/d) and carbohydrate (8.9% at 30 mt-24 h/d), with no significant changes in lipid; increased the chlorophyll a (38.9% at 60 mT, 1 h/d) and b (65% at 30 mT, 1 h/d) and carotenoids (57.8% at 30 mT, 1 h/d) depending on cultivation time	[25]
Microalga (*Chlorella fusca* LEB 111)	15-day-old plants	30 mT or 60 mT at 24 h or 1 h/day	Increased the content of protein (6% at 30 mT,1 h/d) and carbohydrates (25% at 60 mT, 24 h/d) and decreased the lipid content (23% at 60 mT)	[22]
Microalga (*Chlorella fusca* LEB 111)	15-day-old plants	30 mT or 60 mT SMF, for 24 h or 1 h/day	Increased the content of protein (9% at 30 mT, 24 h/d) and carbohydrates (45% at 60 mT, 24 h/d) and decreased the lipid content (15% at 30 mT-24 h) and biomass concentration (13% at 1 h)	[33]
Microalga (*Chlorella pyrenoidosa* (FACHB-9)	6-day-old plants	Irrigated wastewater treatment with SMF 0.5 T for 3 h/day	Increased the lipid productivity (10%) and biomass productivity (12%) and chlorophyll content (ap. 27%)	[137]
Paulownia (Tomentosa and fortunei)	28-day-old plants	2.9–4.8 mT SMF for 2.2, 6.6, or 19.8 s to seedlings	Increased the chlorophyll content of a (19–71%), b (6.5–30%), and total (6.5–53%) depending on variety at 19.8 s	[135]
Soybean (*Glycine max* L. Merrill J 357)	28-day-old plants	2.9–4.6 mT SMF at 2.2 and 19.8 s to seeds	Increased the content of chlorophyll a (21%), b (13%), and total 18%) at 2.2 s	[119]
Soybean (*Glycine max* L. Merrill)	Approx. 15-day-old plants	20 and 30 mT SMF for 5 days, 5 h/d	Increased the content of ferrous (5–28%) total iron (100%), ferritin (ap. 40% for 2 d), iron chelating activity (50% for 2 d) at 30 mT, contrary at 20 mT MF	[64]
Soybean (from Ayyub Agriculture Research Institute)	Seedling of early growth stage	Pre-treatment of SMF 50, 75, and 100 mT for 3 and 5 min to seeds	Increased over 2 times the content of chlorophyll a, b (highest at 75 mT-3 min) and proline (high at 75–100 mT-3 min) soluble sugar (high at 50–100 mT-3 min), and protein (50–100 mT-3–5 min)	[20]
Soybean (*Glycine max* L. Merrill)	45-day-old plants (roots, leaves)	Pre-treatment of SMF 200 mT for 1 h to seeds	Increased the content of total biomass accumulation by 105%, total chlorophyll (26%), rate of photosynthesis (22%), hemichromes (29%), leghaemoglobin (63%)	[44]
Soybean (*Glycine max* L.) variety JS-335)	45-day-old plants	Pre-treatment of 200 mT SMF for 1 h to seeds	Increased the total chlorophyll content (ap. 40%)	[48]
Soybean (*Glycine max)* var. JS-335	45-day-old plants	Pre-treatment of 200 mT SMF for 1 h to seeds	Increased the content of DNA (38%), RNA (17%), and protein (92%), and decreased chlorophyll a/b content (ap. 5%) in leaves	[59]
Soybean (*Glycine max)* var. JS-335)	45-day-old plants	Pre-treatment of 200 mT SMF for 1 h to seeds	Increased the content of total chlorophyll (35%) and carotenoids (24%)	[51]
Soybean (*Glycine max* L. Merrill), cv. Abelina	8-day-old seedlings	Pre-treatment of SMF 250 mT and 500 mT for 3 and 12 min to seeds	Increased the chlorophyll content of a (64%) and b (81%) and carotenoids (364%); highest value at 250 mT for 3 min	[5]
Strawberry: (Camarosa), tomato (Micro-Tom)	45-day-old plants	Irrigated magnetic water by SMF of max 60 mT used during the growing season	Increased the content of chlorophyll a, b (255.9–345.4%) for strawberries and (99.1–108.4%) for tomatoes; increased the mineral content of Mg, Ca, Fe, K, P, and Na in roots from 23.1%(Ca)-184.8% (Fe) for strawberries and from 12.7% (Mg)-84.3% (Fe) for tomatoes	[32]
Sweet pepper (*Capsicum annuum* L.)	90-day-old plants	Pre-treatment of max SMF of 60 mT by seeds or water passing through the magnetic funnel	Increased the content of chlorophyll a (12%) and b (21%), carotenoids (3%), and mineral of P (7%) in leaves for seed+ magnetised water	[136]
Tomato (*Lycopersicum esculentum* L. cv. Strain B)	70-day-old plants	Pre-treatment of max SMF of 50 mT by seeds or water passing through the magnetic funnel	Increased the content of chlorophyll a (28%), b (35%), total (30%), and carotenoids (25%); higher value for magnetised water	[96]
Tree seedlings (*Robinia pseudoacacia*)	60-day-old seedlings	10 mT (69 Hz) MF at 0.5–8 h daily	Increased the content of chlorophyll a by 40% for 0.5 = 1 h exposure time and decreased the nucleic acid level in leaves by ap. 36%	[145]
Wheat (*Triticum aestivum* L. cvs. Tekirdag and Selimiye)	28-day-old cultivars	Pre-treatment of SMF 2.9–4.7 mT at 2.2–19.8 s	Increased the content of chlorophyll a (24%, 32%) and b (70%, 75%), and carotenoids (42%, 33%) for cultivars (Selimiye, Tekirdag) at 2.2 s	[107]
Wheat (*Triticum aestivum* L. cv. Kavir)	Approx. 4-day-old seedlings	30 mT SMF for 4 days, each 5 h of germinated seeds	Increased the proline content (29%) and decreased content of fructans (ap.20%)	[108]
Wheat (*Triticum aestivum* L. cv. Kavir)	Approx. 3-month-old plants	30 mT SMF for 4 days, each 5 h of plants before harvest	Increased ferritin content of shoots by 30%, Fe-bound to total protein content in shoots (40%) and seeds (30%)	[140]
Wheat (Kharchia 65 and HD 2967)	30-day-old plants	Pre-treatment of SMF of 50 mT for 2 h to seeds	Increased the chlorophyll content of a (4–6%) and b (12.5–16%) depending on the variety	[31]
Wheat (*Triticum aestivum* L. cv. Giza 168)	100-day-old plants	Pre-treatment of max SMF of 50 mT by seeds or water passing through the magnetic funnel	Increased the content of total chlorophyll (106%) and carotenoids (40%) for magnetised water and carbohydrate (16%) and protein (10–13%) in grains for magnetised seeds and water; increased the mineral content of N, P, K, Ca, Mg, Fe, Mn, and Cu (338–514%) higher value for roots and magnetised water	[35]

**Table 5:** Effects of MFs on gene expression.

Variety	Plant Species	Method	Effect	Reference
*Arabidopsis thaliana *(L.) Heynh	5-day-old seedlings	0–188 μT for 120 h of plant growing in darkness-or under red or blue light	Increased the transcript levels of the genes rbcl under red (about 4-fold) and blue light (in the peak 10-fold) at about 50 μT, gene pal4 for Ler ecotype (red light), and gene rbcl under blue light for cry1cry2 ecotype; decreased genes cab4 (about 60–100 μT), pal4 and ef1 over 50 μT under blue light for Ler ecotype	[62]
*Arabidopsis thaliana* (L.)	7-day-old plants	With different directions of 300 mT or 600 mT within after 3 d of seed germination to 7 days of growing	Significantly downregulated 85% of the total genes in roots; increased the transgenic line expressing the β-glucuronidase expression domain by 54%	[27]
*Arabidopsis thaliana *(L.)	9-day-old plants	14 T SMF for 2.5 h and 21 T for 2.5 and 6.5 h to 21 days-old plants	Induced the expression of the Adh/GUS transgene in the roots and leaves at over 15 T, and 114 genes were differentially expressed to a degree greater than 2.5-fold	[128]
*Zea mays* (L.)	7–10-day-old plants	Pre-treatment of SMF 3 and 10 mT for 4 h of seeds	Increased the meiotic parameters of total chiasmata, intercalary chiasmata, ring bivalents from 23% to 47% at 3 mT and disorganised chromosome, Anaphase-laggards and -I bridges, micronuclei, clumping, and intertilepollen from 2.6-fold to over 10-fold at 3 and 10 mT	[143]
Tomato seeds var. Pusa Rohini	Germinating seeds of 12 h	Pre-treatment of 100 mT SMF for 30 min	Increased the genes involved in H_2_O_2_ synthesis (AO, SOD1, SOD9) by 21.7-, 2.3-, and 5-fold, respectively, in signalling (ArcA2) by 5.7-fold and in scavenging (MT1) by 14.4-fold and genes involved in ABA deactivation (ABA-H) by 2.8-fold	[57]
Tobacco (*Nicotiana tabacum* L. cv. Burley 21)	Plant cells	0.2 mT SMF up to 24 h with an exposure time of 3–24 h	Increased the transcript accumulation of CDKA (ap. 100% after 8 h), CycD (p. 500% at 8 h), Rbp (ap. 300% for 3–6 h), E2F (300–500% for 3–8 h), and p21 (150–700% for 3–12 h), and TA (150–500% for 3–12 h)	[63]
Soybean (*Glycine max* L. Merrill)	Approx. 15-day-old plants	20 and 30 mT SMF for 5 days, 5 h/d	Increased the expression of CAT gene (ap.38%), ferritin gene (ap.28% at 2 d), and Fe transporter gene (ap.60%) at 30 mT, contrary at 20 mT MF	[64]
Soybean (*Glycine max* L.) variety JS-335)	5-day-old seedlings	200 mT SMF for 1 h to seeds	Increased the genes expression of alpha-amylase (80–110%), nitric oxide synthase (1150%), and nitrate reductase (300%)	[42]

## Data Availability

Not applicable.
